# Adipose-derived mesenchymal stem cell therapy for connective tissue diseases and complications

**DOI:** 10.1186/s41232-024-00348-z

**Published:** 2024-07-19

**Authors:** Takuya Kotani, Takashi Saito, Takayasu Suzuka, Shogo Matsuda

**Affiliations:** 1 Department of Internal Medicine (IV), Division of Rheumatology, Osaka Medical and Pharmaceutical University, Daigaku-Machi 2-7, Takatsuki, Osaka 569-8686 Japan; 2 Department of Legal Medicine, Osaka Medical College, Takatsuki, Osaka Japan

**Keywords:** Mesenchymal stem cells, Adipose-derived stem cells, Connective tissue disease, Interstitial lung disease

## Abstract

Mesenchymal stem cells (MSCs) may be effective in treating connective tissue disease and associated organ damage, leveraging their anti-inflammatory and immunoregulatory effects. Moreover, MSCs may possess the ability to produce antiapoptotic, proliferative, growth, angiogenic, and antifibrotic factors. Among MSCs, adipose-derived MSCs (ASCs) stand out for their relative ease of harvesting and abundance. Additionally, studies have indicated that compared with bone marrow-derived MSCs, ASCs have superior immunomodulatory, proangiogenic, antiapoptotic, and antioxidative properties. However, relatively few reviews have focused on the efficacy of ASC therapy in treating connective tissue disease (CTD) and interstitial lung disease (ILD). Therefore, this review aims to evaluate evidence from preclinical studies that investigate the effectiveness of MSC therapy, specifically ASC therapy, in managing CTD and ILD. Moreover, we explore the outcomes of documented clinical trials. We also introduce an innovative approach involving the utilization of pharmacologically primed ASCs in the CTD model to address the current challenges associated with ASC therapy.

## Background

Recent advances have transformed the treatment landscape of connective tissue disease (CTD), resulting in the availability of various treatment options. For example, the treatment of rheumatoid arthritis (RA) now focuses on achieving remission by combining biological disease-modifying antirheumatic drugs (DMARDs) and targeted synthetic DMARDs with conventional synthetic DMARDs, including methotrexate [1]. For systemic lupus erythematosus (SLE), conventional immunosuppressants, including cyclophosphamide (CYC), azathioprine, and cyclosporine, alongside novel immunosuppressants such as tacrolimus and mycophenolate mofetil (MMF), have demonstrated efficacy in treating lupus nephritis and organ damage associated with SLE [2]. Belimumab—a monoclonal antibody targeting soluble B lymphocyte stimulator—is also effective in suppressing disease activity in SLE and serves as a maintenance therapy for this disease [3]. Advancements in novel immunosuppressants and biological products have improved disease control and CTD prognosis. However, challenges such as the risks of side effects and opportunistic infection owing to immunosuppression persist. Furthermore, effective treatments for many types of CTD, such as systemic sclerosis (SSc), remain few.

CTD is associated with various types of organ damage, with interstitial lung disease (ILD) being particularly important due to its significant impact on patient prognosis. Progressive ILD associated with dermatomyositis/polymyositis, progressive ILD linked to SSc, acute exacerbation of ILD correlated with RA, and comparable conditions present therapeutic challenges and are associated with a poor prognosis [4]. Treatment for these progressive CTD-ILDs typically requires a combination of corticosteroids and immunosuppressive agents, including calcineurin inhibitors, CYC, and MMF, among others [4, 5]. However, numerous cases continue to progress despite the advances, leading to respiratory failure and, ultimately, mortality. Additionally, the prolonged and high-dose utilization of these immunosuppressants raises concerns regarding infections and potential side effects. Recently, pirfenidone and nintedanib have been employed to inhibit fibrotic progression in ILD; however, their efficacy remains limited [5, 6]. Therefore, developing more effective and safer treatments for refractory CTD and complicating ILD, considering their efficacy and safety, is crucial.

Mesenchymal stem cells (MSCs) are being explored extensively in the field of regenerative medicine, given their ability to differentiate into various mesenchymal cells, including osteoblasts, adipocytes, myocytes, and chondrocytes [7, 8]. Moreover, MSCs exhibit antiapoptotic, anti-inflammatory, and antifibrotic effects, along with their ability to modulate the immune response and modify the microenvironment at the engraftment site [7, 9–11]. Thus, the current focus involves investigating the efficacy of MSC therapy for inflammatory and autoimmune diseases. Additionally, MSCs can serve as allografts owing to their low expression levels of human leukocyte antigen (HLA) class I and II [12, 13], and they are well-tolerated when administered intravenously [9]. While research on MSCs has primarily focused on those harvested from the bone marrow, recent studies indicate that these cells can also be harvested from various other tissues, including the cord blood, placenta, and adipose tissue. Adipose tissue specifically contains substantial numbers of MSCs, with subcutaneous adipose tissue being easily accessible. Consequently, adipose-derived mesenchymal stem cells (ASCs) are currently gaining much attention.

Therefore, this review aims to examine evidence from preclinical studies that investigated the efficacy of MSC therapy, particularly focusing on ASC therapy, in managing CTD and ILD. Additionally, we explored the findings of documented clinical trials. Furthermore, this review aims to introduce an innovative approach involving the use of pharmacologically primed ASCs in the CTD model. The study findings could offer a potential strategy to prevent current challenges associated with ASC therapy.

## Immunomodulatory effects of MSCs

MSCs modulate the activities of various immune cells, including lymphocytes, monocytes, and neutrocytes. Furthermore, MSCs inhibit cyclin D2 expression, leading to cell cycle arrest at the G0/G1 phase [14]. They also inhibit the proliferation of CD4^+^ and CD8^+^ T cells, including memory and naïve T cells [15]. The effect on T cells is mediated by specific factors, namely transforming growth factor-β1 (TGF-β1), prostaglandin E2 (PGE2), and indoleamine 2,3 dioxygenase, which are generated by MSCs [16]. Other factors have been observed to mediate the effect of MSCs on T cells. These include IL-6, hepatocyte growth factor (HGF), heme oxygenase 1, HLA-G5, interleukin 1 receptor antagonist, and soluble TNF-receptor 1 [17–20]. MSCs induce anergy by inhibiting proinflammatory cytokines, including IFN-γ, TNF-α, and IL-17, while simultaneously elevating the expression of IL-10 and IL-4 [16, 21, 22]. In addition, inducible nitric oxide synthase, which triggers differentiation into Th2-type T cells and regulatory T cells (Tregs), exhibits cytotoxic effects on T cells and natural killer (NK) cells. Additionally, MSCs suppress the function of Th17-type T cells through cell contact via the programmed death-1/programmed death ligand-1 pathway [23]. Soluble factors produced by MSCs, including PGE2 and TGF-β1, influence the proliferative capacity and/or cytotoxicity of NK cells through direct and indirect mechanisms [24]. The TNF-α-stimulated gene 6 protein (TSG-6), secreted by MSCs, exerts anti-inflammatory effects in macrophages by attenuating TLR2/NF-κB signaling [25].

MSCs inhibit the differentiation of T cell-dependent B cells into plasma cells by suppressing the activity of CD4+ T cells. Furthermore, MSCs directly inhibit the proliferation, differentiation into plasma cells, and chemotaxis [26], consequently exerting an indirect suppression on T cell activation [27]. In addition, MSCs inhibit the differentiation, maturation, and activity of dendritic cells while inducing differentiation into M2 macrophages [28–30]. PGE2 and IL-6—produced by MSCs—induce the secretion of IL-10 by M2 macrophages, consequently inhibiting the neutrophil oxidative burst [31].

The trophic effect of MSCs is predominantly attributed to antiapoptotic, proliferative, growth, and angiogenic factors. Besides their antiapoptotic and angiogenic effects, MSCs prevent fibrosis by promoting the secretion of HFG and matrix metalloproteinases (MMPs) while concurrently downregulating collagen synthesis [32]. Exosomes released by MSCs encompass proteins, messenger RNA (mRNA), and micro RNA, crucial factors that significantly contribute to the trophic effect of MSCs [32].

**Fig. 1:**
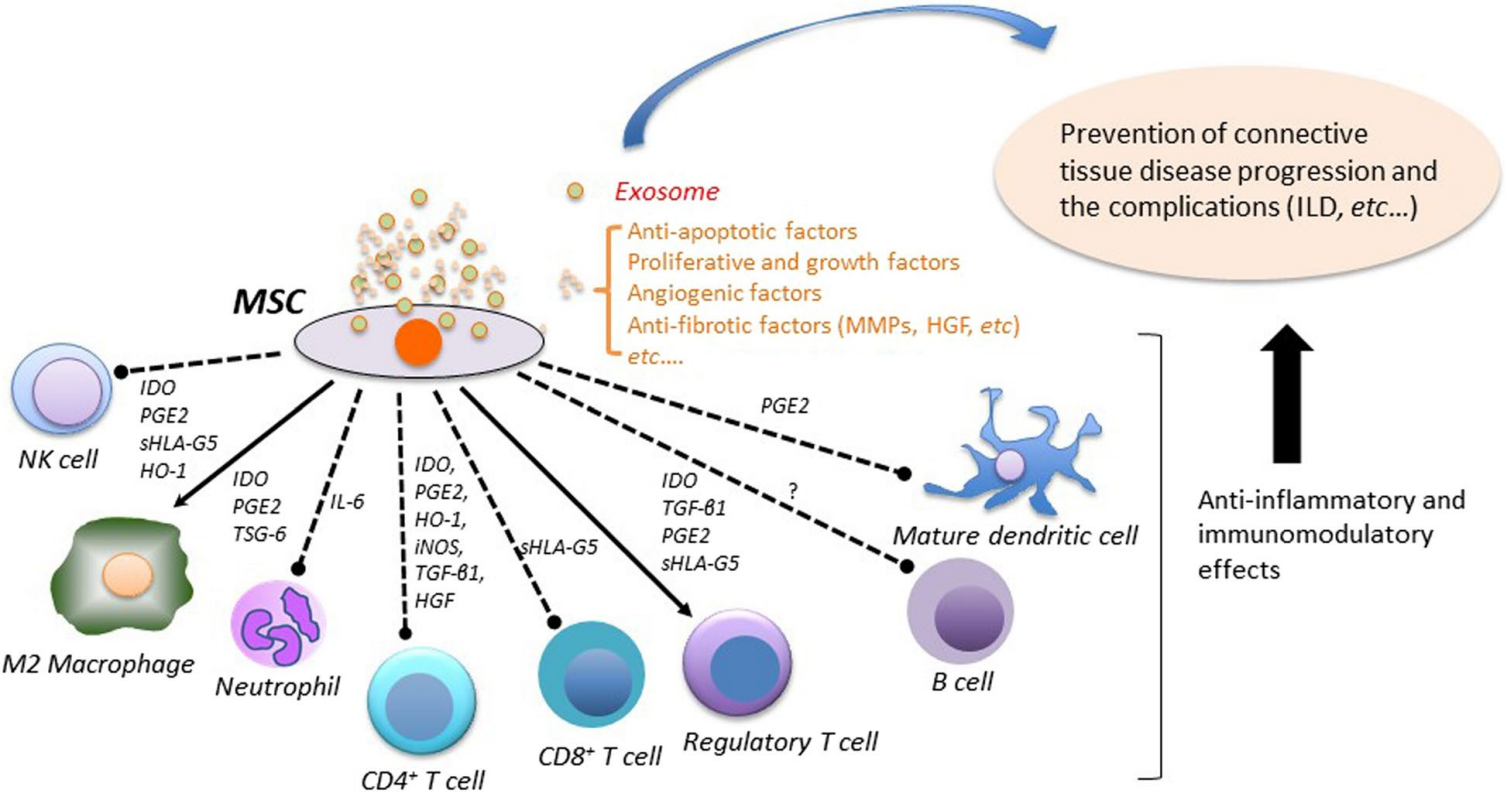
Schematic overview of anti-inflammatory, immunoregulatory effects, and other beneficial factors for preventing disease progression of connective tissue diseases and complications. MSCs exert a multifaceted suppressive effect on various immune cells implicated in CTDs. By inhibiting the proliferation and effector functions of CD4+ and CD8+ T cells, including memory and naïve subsets, MSCs wield their influence through specific factors such as TGF-β1, PGE2, HO-1, iNOS, sHLA-G5, and IDO. This induction of T cell anergy, suppression of proinflammatory cytokines, and elevation of anti-inflammatory cytokines collectively contribute to immune regulation. Furthermore, MSCs modulate the function of regulatory T cells, fostering their differentiation alongside Th2-type T cells, thus further dampening immune responses. Not only do MSCs hinder the differentiation, maturation, and function of dendritic cells, but they also steer the polarization of M2 macrophages, inducing an anti-inflammatory milieu. Through the secretion of soluble factors such as IDO1, PGE2, sHLA-G5, HO-1, and TSG-6, MSCs exert influence on the activity of NK cells and macrophages, enhancing their anti-inflammatory properties. PGE2 and IL-6 from MSCs prompt M2 macrophages to release IL-10, which, in turn, suppresses neutrophil oxidative burst. MSCs further impede CD4+ T cell activity, thwarting T cell-driven B cell differentiation into plasma cells while directly curtailing plasma cell functions. Moreover, through the secretion of HGF and matrix MMPs, along with the downregulation of collagen synthesis, MSCs impede fibrosis. Their trophic effects are significantly augmented by the encapsulation of various bioactive molecules within exosomes released by MSCs. In summary, the collective anti-inflammatory, immunoregulatory, and trophic actions of MSCs position them as highly promising therapeutic candidates for CTDs and their associated complications. MSCs mesenchymal stem cells, CTDs connective tissue diseases, MMPs matrix metalloproteinases, NK natural killer, IDO indoleamine 2,3 dioxygenase, PGE2 prostaglandin E2, TSG-6 TNF-α-stimulated gene 6 protein, sHLA-G5 soluble human leukocyte antigen G5, HO-1 heme oxygenase 1, TGF-β1 transforming growth factor-β1, IL-6 interleukin-6, iNOS inducible nitric oxide synthase, HGF hepatocyte growth factor, ILD interstitial lung disease. Solid line indicates promotion, dotted line indicates suppression

The anti-inflammatory and immunoregulatory effects of MSCs and their capacity to produce antiapoptotic, proliferative, growth, angiogenic, and antifibrotic factors such as MMPs and HGF render MSC therapy an appealing treatment option for CTD and its associated organ involvement (Fig 1).

## Advantages of ASCs compared to other types of MSCs, including bone marrow-derived MSCs and umbilical cord blood-derived MSCs

MSCs exhibit remarkable abilities for self-replication and self-renewal. As adult stem cells are distributed throughout the body, MSCs can be harvested from various sources, including the bone marrow, cord blood, adipose tissue, and placenta. Thus, the utilization of MSCs is associated with fewer ethical concerns compared to that of embryonic stem cells. In addition, compared to induced pluripotent stem cells, MSCs are considered safer to use since there is no requirement for gene transfers.

ASCs are fibroblast-like cells resembling MSCs harvested from other tissues, with differences in cell surface characteristics and gene expression [33]. However, the physiological significance of these differences remains unclear. Bone marrow-derived MSCs (BM-MSCs) constitute only 0.001–0.01% of all nucleated cells [34]. Additionally, studies suggest that the proliferation rate of harvested MSCs tends to decelerate with aging [35]. Therefore, harvesting a sufficient quantity of MSCs for therapy from the bone marrow poses a challenge. Conversely, the number of ASCs that can be harvested from 1 g of adipose tissue (5×10^3^ cells/g) is 500 times greater than that of BM-MSCs for the equivalent amount of the bone marrow [34]. Additionally, ASCs exhibit a faster proliferation rate than BM-MSCs. Therefore, harvesting the required number of ASCs is relatively easy. Regarding immunomodulatory effects, ASCs surpass other types of MSCs, including BM-MSCs, in suppressing the proliferation of T cells, inhibiting the activation and immunoglobulin production of B cells, and suppressing monocyte differentiation into mature dendritic cells [36–40]. Furthermore, ASCs exhibit greater potency than other MSCs based on their proangiogenic, antiapoptotic, and antioxidative effects [41–43]. Collectively, these findings indicate that ASCs may be more effective in suppressing immune response compared to other types of MSCs, including BM-MSCs.

While both ASCs and umbilical cord blood-derived MSCs (UC-MSCs) exhibit significant immunomodulatory properties, current literature delineates several distinct advantages of ASCs over UC-MSCs. Firstly, ASCs are sourced from the adipose tissue, which is not only more abundant but also more readily accessible than the umbilical cord blood, where availability may be constrained [44–46]. Secondly, ASCs display enhanced multipotency and differentiation potential. Empirical evidence suggests that ASCs possess a superior ability to differentiate into adipocytes, osteoblasts, and neuronal cells, thus offering broader therapeutic applications than UC-MSCs [46, 47]. Thirdly, ASCs demonstrate more potent immunomodulatory effects than UC-MSCs. Comparative studies indicate that ASCs may have a more pronounced cytokine secretion profile, significantly impacting immune responses [45, 46]. Fourthly, analyses of proliferation and anti-apoptotic capacities revealed no significant differences between ASCs and UC-MSCs, indicating that both cell types exhibit comparable growth and survival capabilities [46]. In summary, the advantages of ASCs over UC-MSCs, particularly their more accessible source, enhanced multipotency, differentiation potential, and stronger immunomodulatory effects, while maintaining comparable proliferative and anti-apoptotic abilities, position them as preferable candidates for applications in tissue engineering and regenerative medicine.

Table 1 shows a comparison of characteristics among ASCs, BM-MSCs, and UC-MSCs.

**Table 1:** Comparison of the characteristics between ASCs, BM-MSCs, and UC-MSCs

Characteristic	ASCs	BM-MSCs	UC-MSCs
Source	Adipose tissue	Bone marrow	Umbilical cord
Collection method	Liposuction	Bone marrow aspiration	Non-invasive collection at birth
Invasiveness of collection	Less invasive	More invasive	Less invasive
Cell yield	High	Low	Variable, generally lower than ASCs
Isolation procedure	Enzymatic digestion	Density gradient centrifugation	Typical density gradient centrifugation
Proliferation rate	High	Low	Moderate, typically lower than ASCs but higher than BM-MSCs
Differentiation potential	High adipogenic; moderate in other lineages	High osteogenic; moderate in other lineages	Broad, less lineage-specific than ASCs or BM-MSCs
Immunomodulatory effects	High; includes cytokine secretion, T cell and B cell modulation	Moderate; less potent than ASCs	Similar to or less than ASCs
Anti-inflammatory effects	High; includes suppression of pro-inflammatory cytokines	Moderate; less potent than ASCs	Similar to ASCs, variable potency
Angiogenic effects	High; includes secretion of VEGF, FGF, etc.	Moderate; less potent than ASCs	Similar to ASCs, variable
Anti-apoptotic effects	High; includes secretion of Bcl-2, Bcl-xL, etc.	Moderate; less potent than ASCs	Similar to or less than ASCs

*ASCs* adipose-derived mesenchymal stem cells, *BM-MSCs* bone marrow derived mesenchymal stem cells, *UC-MSCs* umbilical cord blood-derived mesenchymal stem cells, *VGEF* vascular endothelial growth factor, *FGF* fibroblast growth factor, *Bcl-2* B-cell lymphoma-2, *Bcl-xL* B cell lymphoma like X, long variant

## MSC/AdSC therapy for preclinical models of connective tissue and interstitial lung diseases

### Rheumatoid arthritis model

Studies using the collagen type II-induced arthritis model reported conflicting results. While some studies showed that the systemic administration of MSCs improved arthritis-associated inflammation [48–51], others reported its ineffectiveness or observed worsening of symptoms [52, 53]. The efficacy of MSC therapy depends on factors, including the route, frequency, and timing of administration. Based on the findings of these previous studies, MSC transplantation should precede collagen induction, with short intervals between each administration. Studies reporting improvements after MSC therapy in the collagen type II-induced arthritis model found a reduced incidence of arthritis, lower disease activity score (DAS), an improved balance between pro- and anti-inflammatory cytokines, and reduced pathological scores reflecting the degree of joint destruction. Regarding ASCs, the systemic administration of human ASCs in DBA/1 mice induced with collagen-induced arthritis notably alleviated arthritis severity. This therapeutic effect originated from the suppression of two pivotal disease components: the Th1-driven autoimmune response and the associated inflammatory reaction [50]. Ueyama et al. reported reduced intra-articular inflammation and significant cartilage regeneration in SKG mice through the local injection of single-cell mouse ADSCs and three-dimensionally cultured ADSC spheroids [51].

### Systemic lupus erythematosus model

The therapeutic effectiveness of MSCs in the SLE mouse model shows variability, influenced by factors such as the mouse strain and origin or condition of the MSCs. Upon transplantation of BM-MSCs into MRL/Ipr mice, a decrease in serum levels of anti-ds-DNA antibodies, antinuclear antibodies, and immunoglobulins was observed. Concurrently, kidney function improved, complement 3 levels decreased, and glomerular IgG deposition lessened [54, 55]. Conversely, the systemic administration of BM-MSCs into NZB/W F1 mice was reported ineffective and, in some cases, exacerbating the disease [56, 57]. Gu et al. utilized MRL/Ipr and NZB/W F1 mouse models, demonstrating that BM-MSCs derived from healthy young and diseased mice ameliorated SLE-like disease while reducing T and B lymphocyte levels in the spleen. However, BM-MSCs derived from old NZB/W F1 mice did not reduce spleen weight, IgG deposition, kidney disease, or inflammation in the renal interstitium [58]. In contrast to BM-MSCs, Gu et al. utilized umbilical cord blood-derived MSCs (UC-MSCs) in MRL/lpr mice. They demonstrated a dose-dependent improvement in lupus nephritis-associated [59]. Chang et al., using NZB/W F1 mice, demonstrated that the transplantation of human UC-MSCs significantly delayed the onset of proteinuria, reduced serum anti-ds-DNA antibody levels, mitigated renal dysfunction, and prolonged mouse survival [60]. Zhang et al. also reported marked improvement in nephritis in MRL/lpr mice following weekly systemic administration of ASCs at a dose of 1×10^6^ cells for eight consecutive weeks. They observed a substantial decrease in Th17 cells within the spleen and a significant increase in Treg cells [61]. Choi et al. also reported that long-term repeated administration of human ASCs improved SLE symptoms in NZB/W F1 mice [62]. Mice-administered ASCs exhibited a higher survival rate than those in the control group, demonstrating improvements in pathological and serological abnormalities, enhanced immune functions, and reduced proteinuria incidence. This administration also resulted in a notable decrease in serum anti-ds-DNA antibody and urea nitrogen levels, alongside increased serum granulocyte–macrophage colony-stimulating factor, IL-4, and IL-10. Furthermore, a significant rise in Treg cell proportion was observed in the spleen of ASC-administered mice.

### Systemic sclerosis model

In a hypochlorous acid (HOCl)-induced SSc mouse model, characterized by induced fibrosis in the skin and lung through HOCl, a systemic administration of 2.5×10^5^ BM-MSCs was conducted thrice [63]. The result showed that BM-MSC administration reduced the deposition of all collagen in the skin and lung tissues, and it downregulated the expression of αSMA and TGF-β1 mRNAs. Additionally, the level of anti-Scl-70 antibodies in the serum decreased, alongside a reduction in macrophage infiltration and T cells in the skin. The treatment also led to an improvement in tissue remodeling. Moreover, BM-MSCs did not accumulate in the skin, and they were cleared from the lungs within several days. Similar results were observed in allotransplantation, xenotransplantation, and syngeneic transplantation. The authors also compared the treatment effects of human BM-MSCs and ASCs in HOCl-SSc mice, revealing that ASCs were more effective in suppressing dermal thickening. Furthermore, ASCs significantly downregulated mRNA expression for inflammatory cytokines and factors associated with tissue remodeling in the skin and lung tissues compared to BM-MSCs [64]. The subcutaneous administration of autologous ASCs improved dermal fibrosis in the bleomycin-induced skin fibrosis mouse model [65]. Furthermore, intravenous administration of allogeneic ASCs attenuated skin fibrosis in the bleomycin-induced scleroderma and Scl-cGVHD mouse models [66, 67].

### Interstitial lung disease model

An ILD model utilizing BLM-induced lung fibrosis has been employed to evaluate the efficacy of MSCs. A review consolidating 36 preclinical trials of MSC therapy for BLM-induced pulmonary fibrosis models has been published [68]. In these studies, the therapy consistently demonstrated a reduction in various aspects of BLM-induced pulmonary damage, including tissue inflammation, inflammatory cell infiltration, inflammatory cytokine expression, extracellular matrix production, and collagen deposition. This collectively demonstrated an improvement in lung fibrosis scores. Several studies have highlighted the effectiveness of intraperitoneally or intravenously administered ASCs and condition medium from ASCs in inhibiting pulmonary fibrosis in BLM-induced ILD mice [69–72].

Specific animal models for ILD associated with CTD have not yet been established. The ILD induced by BLM administration through the airway triggers localized inflammatory and fibrotic changes around the peribronchiolar region. However, these alterations do not precisely mimic the pathological manifestations of ILD associated with CTD. Conversely, continuous subcutaneous BLM administration via osmotic minipump results in inflammatory and fibrotic changes on the pleural side. The lesion distribution originating from these changes is comparable to that observed in ILD associated with CTD [73]. Consequently, we investigated the therapeutic effect of intravenous ASC transplantation on a BLM-induced ILD mice model exhibiting diffuse lesions on the pleural side. The results showed that ASCs inhibited inflammation and fibrosis in the lungs in a manner dependent on the number of administered cells [74].

The abovementioned MSCs/ASCs therapies for preclinical models of CTDs and ILD are presented in Table 2.

**Table 2:** MSCs/ASCs therapy for preclinical models of CTDs and ILD

Target diseases	Disease model	Animal species	Type of MSCs	Cell number	Administration method	Number of doses	Dosage interval	Remarkable findings	Ref
RA	Collagen-induced arthritis	Mouse	Mouse BM-MSCs	5×10^6^ cells	IP	Single	-	Improved arthritis score, suppressed joint destruction, decreased TNF-a, increased Treg	48
RA	Collagen-induced arthritis	Mouse	Human UC-MSCs	1×10^6^ cells	IP	Five	1day	Improved arthritis score, suppressed joint destruction, decreased TNF-a, IL-6, increased Treg, IL-10	49
RA	Collagen-induced arthritis	Mouse	Human ASCs	1×10^6^ cells	IP	Five	1day	Improved arthritis score, decreased TNF-a, IL-6, increased Treg, IL-10	50
RA	Collagen-induced arthritis	Mouse	Mouse ASCs	1.5×10^4^ cells	IA	Single	-	Improved arthritis score, pathology scores for synovitis and cartilage injury, suppressed infiltration of CD4 T cell and macrophage	51
RA	Collagen-induced arthritis	Mouse	Flk-1+Mouse BM-MSCs	1–2×10^6^ cells	IP	Single	-	Exacerbated arthritis score, increased serum IL-6	52
RA	Collagen-induced arthritis	Mouse	Mouse EC-MSCs	4×10^6^ cells	IV	Single	-	Exacerbated arthritis score	53
SLE	MRL/lpr mice	Mouse	Mouse BM-MSCs	0.1×10^6^ cells/10g body weight	IV	Single	-	Improved renal damage (reduced anti-dsDNA antibodies IgM and IgG, ANA levels)	54
SLE	MRL/lpr mice	Mouse	Human BM-MSCs	1×10^6^ cells	IV	Single	-	Decreased anti-dsDNA antibodies, 24-h proteinuria, and CD4+ T cells	55
SLE	(NZB×NZW)/F1 mice	Mouse	Mouse BM-MSCs	1.25×10^6^ cells	IV	Three	1 week	Reduced glomerular immune complex deposition, lymphocytic infiltration, and proliferation	56
SLE	(NZB×NZW)/F1 mice	Mouse	Mouse BM-MSCs	1×10^6^ cells	IP	Single	-	Worsened kidney pathology with increased anti-dsDNA antibody production	57
SLE	(NZB×NZW)/F1 mice, MRL/lpr mice	Mouse	Mouse BM-MSCs	1×10^6^ cells	IV	Single	-	Ameliorated kidney injury, marked by reduced splenic CD3+CD4+ T lymphocytes and CD19+CD21+ B lymphocytes	58
SLE	MRL/lpr mice	Mouse	Human UC-MSCs	1×10^6^ cells	IV	Three	1 week	Decreased 24-h proteinuria, serum creatinine, and anti-dsDNA antibodies, along with reduced renal injury	59
SLE	(NZB×NZW)/F1 mice	Mouse	Human UC-MSCs	1×10^6^ cells	IV	Single	-	Delayed onset of proteinuria, reduced serum anti-dsDNA antibodies, mitigated renal dysfunction, and prolonged survival	60
SLE	MRL/lpr mice	Mouse	Mouse ASCs	1×10^6^ cells	IV	Eight	1 week	Improved 24-h proteinuria, anti-dsDNA antibodies, serum creatinine levels, with reduced Th17 cells and increased Treg cells	61
SLE	(NZB×NZW)/F1 mice	Mouse	Human ASCs	5×10^6^ cells	IV	28	2 weeks	Higher survival rate with improved histologic and serologic abnormalities	62
SSc	HOCl-induced model	Mouse	Mouse BM-MSCs	2.5×10^5^ cells	IV	Single	-	Decreased skin fibrosis, infiltration of CD3+T cell and macrophage, parameters of remodeling and oxidative stress	63
SSc	HOCl-induced model	Mouse	Mouse BM-MSCs, human BM-MSCs, human ASCs	2.5×10^5^ cells	IV	Single	-	Decreased skin fibrosis, infiltration of CD3+ T cells and macrophages, parameters of inflammatory and remodeling	64
SSc	Bleomycin-induced model	Mouse	Mouse ASCs	2×10^6^ cells	SC	Single	-	Decreased skin fibrosis, TGF-b	65
SSc	Bleomycin-induced model	Mouse	Mouse ASCs	5×10^5^ cells	IV	Single	-	Decreased skin fibrosis, profibrotic mRNA, TNF-α, and αv-integrin, suppressed AKT pathway	66
SSc	Bleomycin-induced model/Scl-cGVHD model	Mouse	Mouse ASCs	2×10^5^ cells	IV	Single	-	Decreased skin fibrosis, immune cell infiltration, IL-6, fibrotic cytokines	67
ILD	Bleomycin-induced pulmonary fibrosis	Mouse	Human ASCs	3×10^5^ cells	IP	Four	2 weeks	Ameliorated hyperplasia of club cells (Clara cells) and cuboidal alveolar epithelial cells, along with reduced fibrosis	68
ILD	Bleomycin-induced pulmonary fibrosis	Mouse	Mouse ASCs	5×10^5^ cells	IV	Single	-	Decreased lung fibrosis, MMP-2 activity, oxidative stress, and apoptosis markers	69
ILD	Bleomycin-induced pulmonary fibrosis	Rat	Rat ASCs	1×10^6^ cells	IV	Single	-	Prevention of collagen deposition and lung tissue remodeling	70
ILD	Bleomycin-induced pulmonary fibrosis	Mouse	Mouse ASCs	5×10^6^ cells	IV	Single	-	Reduced collagen deposition and expression of lung fibrosis-related factors	71
ILD	Bleomycin-induced pulmonary fibrosis	Mouse	Mouse ASCs	2.5×10^4^cells, 2.5×10^5^cells	IV	Single	-	Improved survival rate and lung injury in a dose-dependent manner	72

*RA* rheumatoid arthritis, *SLE* systemic lupus erythematosus, *SSc* systemic sclerosis, *ILD* interstitial lung disease, *MSC* mesenchymal stem cell, *ASCs* adipose-derived MSCs , *BM-MSCs* bone marrow-derived MSCs, *EC-MSCs* extraembryonic mesenchymal stem cells, *UC-MSCs* umbilical cord blood-derived mesenchymal stem cells, *IP* intra-peritoneal injection, *IA* intra-articular injection, *IV *intravenous injection, *SC* subcutaneous injection, *TNF-a* tumor necrosis factor-a, *IL* interleukin, *TGF-b* transforming growth factor-β, *MMP-2* matrix metalloproteinase-2, *Treg* regulatory T cells, *Th17* T helper 17 cells, *ANA* antinuclear antibody, *mRNA* messenger RNA

## Pharmacologically primed AdSC therapy in CTD model

### Current challenges in AdSC therapy

Intravenous administration of ASCs presents several associated challenges. A preclinical study found that mice developed pulmonary embolisms following the intravenous administration of a large number of mASCs [75]. Moreover, a clinical study revealed that three patients who were intravenously administered ASCs developed pulmonary embolism [76]. Although minimizing the number of intravenously administered ASCs is preferable, the treatment effect may also be diminished with a low cell count. Elderly individual-derived ASCs typically exhibit poorer proliferative and chemotactic activities than those derived from young individuals [77]. Furthermore, MSCs derived from patients with SLE exhibit characteristics associated with the initial phase of aging and demonstrate reduced function [78], while ASCs derived from patients with scleroderma demonstrate reduced proliferative, metabolic, and chemotactic activities compared to those from healthy individuals [79]. Therefore, treating the potential induction of pulmonary embolism by intravenous ASC administration and improving the functionality of ASCs in elderly patients and those with underlying diseases necessitate the development of novel strategies.

### Previous reports on pharmacologically primed AdSC therapy in the CTD model

To address the challenges associated with ASC treatment, recent studies have explored the therapeutic application of pharmacologically enhanced ASCs in CTD animal models. Kim et al. observed a significant alleviation of symptoms related to graft-versus-host disease in NOD-SCID mice with the administration of MSCs, including ASCs with IFN-γ. This finding implies that the therapeutic effect may be attributed to the induction of IDO expression in MSCs through the IFN-γ-JAK-STAT1 pathway [80]. Zolfaghari et al. demonstrated that TLR3 ligand-primed ASCs using polyinosinic:polycytidylic acid (poly I:C) reduced splenocyte proliferation in vitro. Furthermore, in animal models of adjuvant-induced arthritis, these cells significantly improved clinical and histopathological severity, notably reducing TNF-α and IL-6 levels in serum [81]. Jang et al. reported that metformin enhanced the immunoregulatory effect of ASCs by upregulating STAT1 expression through the AMPK/mTOR pathway in vitro. Administering metformin-treated ASCs markedly improved disease activity, including inflammatory phenotype, glomerulonephritis, proteinuria, and anti-dsDNA IgG antibody production in MRL/lpr mice. Additionally, metformin-treated ASCs inhibited CD4-CD8- T cell proliferation and modulated the Th17/Treg cell ratio [82].

### Heparin-primed ASC therapy in the CTD model

**Fig. 2:**
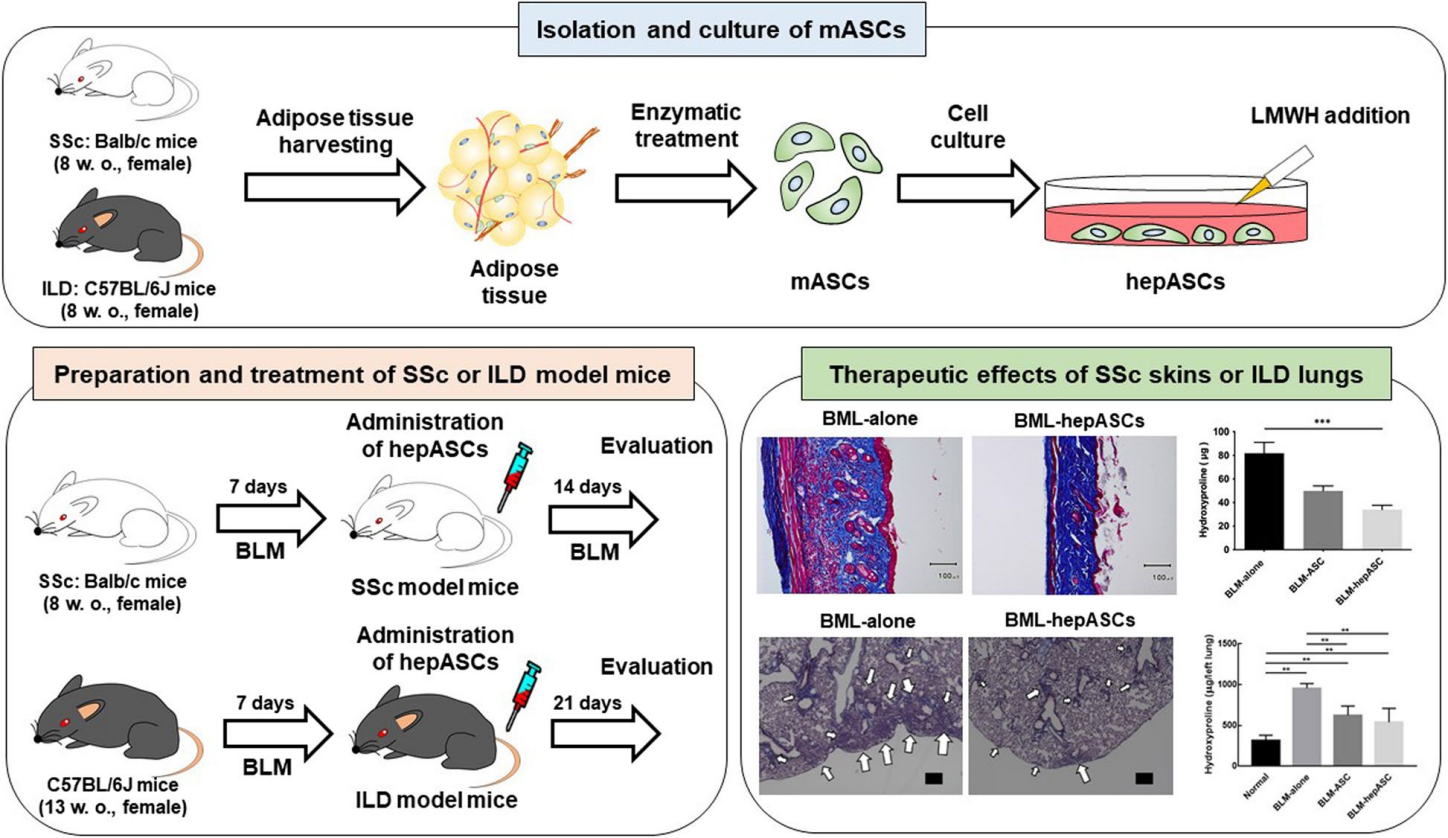
Enhanced techniques for isolation and co-cultivating heparin-enhanced murine adipose-derived stem cells, with details on preparation, treatment protocols, and therapeutic effects for systemic sclerosis and interstitial lung disease model mice. *Isolation and culture of ASCs:* ASCs were isolated from the inguinal adipose tissue of 8-week-old female Balb/c mice (for SSc) and 8-week-old female C57BL/6J mice (for ILD), following euthanasia by cervical dislocation under isoflurane anesthesia. The adipose tissue was washed in PBS, minced, and digested with type I collagenase (1.0 mg/mL in 1% BSA/HBSS (+)) at 37 °C for 30 min. After filtration and centrifugation, the cell pellet was resuspended in erythrocyte lysis buffer, followed by medium addition and centrifugation. The resulting mesenchymal ASCs were cultured at a density of 1×10^6^ cells per 90 mm cell-culture dish and utilized at the third passage. *Preparation and treatment of SSc and ILD model mice:* HepASCs were cultured in DMEM/F-12 with 10% FBS, 1% Pen-Strep, and LMWH. For SSc studies, female BALB/c mice (8 weeks old) received daily subcutaneous injections of 100 μg/100 μL BLM for 21 days. These mice were divided into three groups: BLM alone, BLM with ASCs, and BLM with hepASCs. For ILD studies, female C57BL/6J mice (13 weeks old) were divided into four groups: untreated (normal), BLM alone, BLM with ASCs, and BLM with hepASCs. BLM (3 mg in 100 μL saline) was administered subcutaneously over 7 days. For both studies, cells (1.0×10^5^ for lung, 2.5×10^4^ for skin in 100 μL PBS) or PBS was intravenously injected. Mice were euthanized on day 21 for skin analysis and day 28 for lung analysis, with respective organs harvested for evaluation. *Therapeutic effects of SSc skins and ILD lungs:* intravenously administered hepASCs significantly reduced skin thickness and hydroxyproline content in mice with bleomycin-induced SSc compared to that of the SSc pathological model group. Additionally, hepASCs decreased collagen deposition and hydroxyproline levels in the lungs compared to those in the ILD pathological model group. ASCs adipose-derived mesenchymal stem/stromal cells, LMWH low-molecular-weight heparin, SSc systemic sclerosis, ILD interstitial lung disease, hepASCs ASCs enhanced by LMWH, BLM bleomycin. Arrows indicate collagen deposition area; scale bars in skins and lungs are 300 μm and 100 μm, respectively. Data were analyzed using nonparametric one-way ANOVA, followed by a multiple comparison test. *P < 0.05, **P < 0.01, and ***P < 0.001, a significant difference between the linked groups

Heparin—an inhibitor of antithrombin III and factor Xa used in preventing and treating thrombosis [83]—interacts with various proteins, demonstrating multifaceted efficacy [84]. Heparin stabilizes HGF dimers, thereby promoting the dimerization and activation of the c-Met receptor [85]. It augments fibroblast growth factor and bone morphogenetic protein 4 gene expression, consequently enhancing proliferation and pluripotency in BM-MSCs and embryonic stem cells, respectively [86–88]. Furthermore, heparin stimulates HGF biosynthesis in diverse cell types, including lung fibroblasts, promyelocytic leukemia cells, and umbilical vein endothelial cells [89, 90]. Therefore, we hypothesized that heparin-activated ASCs might exhibit a synergistic beneficial effect on SSc or ILD by promoting anti-inflammatory and antifibrotic responses. We investigated the effect of heparin on ASC functions. We compared the therapeutic effects of heparin-enhanced ASCs (hepASCs) to that of ASCs alone in mouse models of SSc and ILD [91, 92]. Figure 2 shows the procedures for isolating and culturing mASCs, the preparation and treatment of SSc and ILD model mice, and the therapeutic effects of SSc skins and ILD lungs. The results showed that heparin significantly increased ASC numbers and enhanced their migratory, anti-inflammatory, and antifibrotic effects in vitro. Additionally, hepASCs demonstrated increased accumulation in the skin or lung tissues compared to ASCs alone. Moreover, in mice with bleomycin-induced SSc or ILD, intravenously administered hepASCs significantly reduced skin thickness and hydroxyproline content compared to the SSc pathological model group. It also decreased collagen deposition and hydroxyproline levels in the lungs compared to those in the ILD pathological model group.

### Potential of MSCs transplant rejection and activation of the immune system

Several reports show that transplanting human MSCs into mice can trigger transplant rejection despite MSCs’ immunosuppressive properties. Various studies indicate that human MSCs provoke an immune response in mice, contrary to prior beliefs about their immune-privileged status [93–95]. This response is most pronounced in xenogeneic transplantation (human MSCs into mice), followed by allogeneic and syngeneic transplantation [94, 95]. Notably, xenotransplantation results in significant infiltration of leukocytes at the injection site, indicating innate immune system activation [93]. While inevitable for preclinical evaluation, xenotransplantation poses challenges for clinical translation of human MSC therapy. Studies suggest using immunosuppressants, such as dexamethasone and tacrolimus, to manage immune responses [93, 96]. Effective immunosuppression protocols are crucial for successful xenotransplantation experiments. In essence, despite MSCs’ immunosuppressive properties, transplant rejection can occur when transplanting them into mice. Mitigating immune responses through careful immunosuppressant use is thus vital for successful xenotransplantation studies.

## Clinical applications of MSCs/ASCs for CTDs and ILD

Table 3 shows the published major clinical trials of MSCs/ASCs therapy for RA, SLE, SSc, and ILD patients.

**Table 3:** Published major clinical trials of MSCs/ASCs therapy for RA, SLE, SSc, and ILD patients

Target diseases	Type of MSCs	Cell number	Administration method	Number of doses	Dosage interval	Number of patients	Remarkable findings	Ref
RA	UC-MSCs	4×10^7^ cells	IV	Repeated	3–6 months	172	Decreased TNF-α and IL-6; increased regulatory T cells; significant remission	97
RA	UC-MSCs	2.5×10^7^–1×10^8^ cells	IV	Single	-	9	No major toxicity; decreased IL-1β, IL-6, IL-8, TNF-α; improved DAS28 score	98
RA	BM-MSCs	1×10^6^ cells/kg	IV	Single	-	13	Increased FOXP3 expression; increased IL-10 and TGF-β1 levels	99
RA	BM-MSCs	4×10^7^ cells/joint	IA	Single	-	15	Improved WOMAC score, pain-free walking distance, standing time, decreased VAS, time to jelling	100
RA	UC-MSCs	4 × 10^7^ cells/patient	IV	Single	-	64	ESR, CRP, RF, anti-CCP, health index, and DAS28 decreased	101
RA	BM-MSCs	1×10^6^ cells/kg	IV	Single	-	9	Significant decrease in Th17 cells, increased regulatory T cells; DAS28-ESR and VAS scores decreased significantly; no significant change in CRP and anti-CCP levels	102
RA	ASCs	1–4×10^6^ cells/kg	IV	Three	1, 8, and 15 days	53	Well tolerated; no dose-related toxicity; trend for clinical efficacy	103
SLE	UC-MSCs, BM-MSCs	1×10^6^ cells/kg	IV	Single	-	15	Improved SLEDAI score, decreased proteinuria, improved renal function, no serious adverse events	104
SLE	UC-MSCs	1×10^6^/kg	IV	Single	-	16	Improved SLEDAI score, decreased ANA and anti-dsDNA, improved renal function, increased Treg cells, stabilized cytokine balance, no recurrence, no treatment-related deaths	105
SLE	UC-MSCs	1×10^6^/kg	IV	Two	0 and 7 days	40	Well tolerated; improved SLEDAI and BILAG scores, decreased proteinuria; some relapses after 6 months, indicating need for repeated MSCT	106
SLE	UC-MSCs	2×10^8^ cells	IV	Single	-	18	Similar remission rates in MSC and placebo groups, no additional effect over standard immunosuppression	107
SLE	UC-MSCs	1×10^6^/kg	IV	Single	-	21	Significant upregulation of CD1c+ dendritic cells, decreased serum FLT3L, potential immunoregulatory mechanism	108
SLE	UC-MSCs	1×10^6^/kg	IV	Single	-	10	sHLA-G levels negatively correlated with SLEDAI score	109
SLE	UC-MSCs	1×10^7^/kg	IV	Single	-	87	50% complete remission at 4-year follow-up, 94% overall survival rate	110
SLE	ASCs	1×10^8^ cells	Intranasal injection, lymph node injection, IV	IN, LN; single, IV; four	Initial three intravenous transfusions within 1 week Fourth intravenous transfusion 9 months later	1	Improved SLE disease activity; no serious adverse events reported	111
SSc	ASCs	2.5×10^4^ cells	HI	1 to 10 times	NA	62	Significant reduction in mRSS, improvement in digital ulcers, lung function, and mouth opening	112
SSc	ASCs	NA	HI	Single	-	7	Improvement in skin elasticity and tightness, decreased skin thickening	113
SSc	SFV	NA	HI	Single	-	6	Improvement in skin elasticity and tightness. No AEs reported.	114
SSc	SFV	NA	HI	Single	-	12	Significant reduction in mRSS, improvement in hand function, decreased skin thickness	115
SSc	BM-MSCs	1.8–21.6×10^7^/kg	IV	Single	-	5	Improvement in skin condition, reduced fibrosis, minor respiratory tract infection	116
SSc	SVF	3.61×10^6^	HI	Single	-	18	Improvement in hand function, decreased Raynaud's condition score	117
SSc	ASCs	4 to 8×10^6^/ml of HA	HI	Single	-	6	Improvement in skin elasticity and tightness. No AEs reported.	118
SSc	UC-MSCs	1×10^6^/kg	IV	Single	-	14	Improvement in lung function, reduced inflammation markers. Minor respiratory tract infection and diarrhea	119
IPF	BM-MSCs	20, 100, or 200×10^6^ cells	IV	Single	-	9	No treatment-emergent serious adverse events; mild decline in FVC and DLCO by 60 weeks	121
CLAD	BM-MSCs	2×10^6^ cells/kg	IV	Four	Twice weekly for 2 weeks	10	Well tolerated; slowed decline in FEV1; no procedure-related serious adverse events	122
IPF	ASCs-SVF	0.5×10^6^ cells/kg	Endobronchial	Three	Baseline, 6 and 12 months	14	No serious adverse events; stable functional and quality of life parameters	123

*RA* rheumatoid arthritis, *SLE* systemic lupus erythematosus, *SSc* systemic sclerosis, *IPF* idiopathic pulmonary fibrosis, *CLAD* chronic lung allograft dysfunction, *MSC* mesenchymal stem cell, *UC-MSCs* umbilical cord blood-derived MSCs, *ASCs* adipose-derived MSCs, *BM-MSCs* bone marrow-derived MSCs, *SVF* stromal vascular fraction, *HA* hyaluronic acid, *IV* intravenous injection, *IA* intra-articular injection, *HI* hypodermic injection, *TNF-a* tumor necrosis factor-a, *IL* interleukin, *TGF-b* transforming growth factor-β, *FOXP3* forkhead box protein P3, *DAS28 score* disease activity score in 28 joints, *Womac score* Western Ontario and McMaster Universities Osteoarthritis Index, *VAS* visual analogue scale, *SLEDAI score* systemic lupus erythematosus disease activity index, *BILAG score* British isles lupus assessment group, *sHLA-G* soluble human leukocyte antigen-G, *mRSS* modified Rodnan skin score, *ESR* erythrocyte sedimentation rate, *CRP* C-reactive protein, *RF* rheumatoid factor, *CCP* cyclic citrullinated peptide, *ANA* antinuclear antibody, *FLT3L* FLT3 ligand, *FVC* forced vital capacity, *DLCO* diffusing capacity of the lung for carbon monoxide, *MSCT* multi-slice computed tomography, *AE* adverse event

### Rheumatoid arthritis

In an uncontrolled phase I/II study, 136 patients with RA resistant to conventional drug treatments were treated with intravenous administration of 4×10^7^ UC-MSCs. They were followed up for 8 months [97]. The result showed significant improvements in the DAS of RA, health assessment questionnaire, and American College of Rheumatology (ACR) response and an increased frequency of Tregs in the peripheral blood. Patients whose disease activity worsened following the treatment received an additional administration of UC-MSCs after 3 months. At the end of the study, 58% of the patients had achieved ACR20, and none of them experienced severe adverse events. Additionally, reports indicate the potential inhibitory effects of single intravenous administrations of BM-MSCs and UC-MSCs on the disease activity of RA in a limited number of cases [98–102]. In 2017, Álvaro-Gracia et al. conducted a multicenter, non-randomized, single-blinded (double-blinded for efficacy assessment), placebo-controlled phase Ib/IIa study to examine the safety of intravenously administered allogenic ASCs in 53 patients with RA with active disease refractory to at least two administrations of biological DMARDs [103]. These researchers administered 1×10^6^, 2×10^6^, or 4×10^6^ ASCs. The results showed that a higher proportion of patients achieved ACR20 and ACR50 and improved DAS in a dose-dependent manner than those in the placebo group. This study primarily aimed to determine the safety of ASCs, and the findings revealed that the treatment was well-tolerated with no serious side effects, except for one patient who developed lacunar infarction.

### Systemic lupus erythematosus

Studies conducted to date have examined the use of MSCs for treatment-resistant SLE, involving a few to over 80 patients. MSCs employed in these studies were allogeneic BM- or UC-MSCs, administered intravenously. These studies collectively found that MSC therapy was well-tolerated and effective in improving kidney function and disease activity, reducing proteinuria, and reducing anti-ds-DNA antibody levels. MSC transplantation resulted in an elevation in Tregs in the peripheral blood and restored the balance between Th1 and Th2 cytokines [104–110]. One of the representative trials conducted by Wang *et al.* was a multicenter study in which 40 patients with refractory SLE were administered 1×10^6^/kg UC-MSCs on days 0 and 7 [106]. Following a 6-month follow-up, 32.5% of the patients achieved complete remission, while 27.5% attained partial remission. However, 17.5% of the patients experienced recurrence. The dosage of immunosuppressants required was significantly reduced in most patients. Throughout the follow-up period, three patients developed herpes simplex virus infection, and one patient contracted tuberculosis. Furthermore, three patients died during the follow-up. The causes of death included acute cardiac arrest 7 days after MSC administration, severe pulmonary hypertension that manifested 8 months after the treatment, and pulmonary infection. Nonetheless, none of these causes were likely associated with MSC therapy. A reported case described a 9-year-old girl with SLE showed improved disease activity after AdSC administration via one nasal injection, one lymph node injection, and two intravenous injections [111].

### Systemic sclerosis

Based on previous studies on MSC therapy for SSc patients, a systematic review and meta-analysis were conducted to assess the efficacy and safety of MSCs in treating SSc [112–120]. The study encompassed nine clinical trials involving 133 adult patients with SSc up to February 1, 2021. These trials included one case of intravenous BM-MSC administration, two cases of intravenous UC-MSC administration, three cases of subcutaneous stromal vascular fraction administration, and three cases of subcutaneous AdSC administration. MSC therapy significantly reduced the modified Rodnan skin score, digital ulcer count, oral handicap scales, and visual analog scales in patients with SSc. Although a few patients presented with injection site swelling, diarrhea, and joint pain, these issues resolved on their own, and no severe adverse events were observed. Overall, the utilization of MSCs was deemed safe.

### Interstitial lung disease

To date, no clinical studies have specifically examined MSC therapy for ILD associated with CTD. However, studies on MSC therapy for idiopathic pulmonary fibrosis (IPF) exist. Glassberg et al. conducted the first trial assessing BM-MSCs in patients with mild to moderate IPF [121]. They infused BM-MSCs from two donors into nine patients. They observed no severe treatment-related side effects with doses of up to 2×10^8^ cells over 60 weeks. However, two deaths unrelated to the study occurred, and 78% of patients reported non-serious adverse events such as bronchitis and colds. Nevertheless, declines in lung function persisted below the recognized threshold for disease progression, with a 3.0% and 5.4% decrease in predicted FVC and DLCO 5.4% after 60 weeks, respectively. Chambers et al. conducted an open-label, single-center, non-randomized, uncontrolled, phase Ib dose-escalation study involving eight patients with moderately severe but not advanced IPF (FVC ≥50% of predicted normal; DLCO ≥35% of predicted normal) [122]. The patients were administered two intravenous doses of HLA-unmatched placenta-derived MSCs (1×10^6^/kg body weight, *n* = 4; 2×10^6^/kg/kg body weight, *n* = 4). During the 6-month follow-up period, one episode of lingular left lobe consolidation, considered possibly treatment-related, occurred 5 days after the infusion of the lowest cell dose. No other severe side effects attributed to the administration of MSCs were reported. Tzouvelekis et al. conducted an open-label, single-group, non-comparative, phase-Ib clinical trial comprising 14 patients with mild or moderate IPF (FVC >50% of predicted normal; DLCO >35% of predicted normal) [123]. The patients were treated with endobronchial administration of autologous adipose tissue-derived stromal cell-stromal vascular fraction (0.5×10^6^/kg body weight/dose, administered thrice at monthly intervals). No other severe side effects directly attributed to the administration of these cells were reported.

Table 4 presents the current clinical trials identified through the National Library of Medicine website of the National Institutes of Health for RA, SLE, SSc, and ILD (visit: www.clinicaltrials.gov). The number of clinical trials assessing the effectiveness and safety of BM-MSCs and UC-MSCs for CTD and ILD is on the rise. However, studies on AdSCs in this context remain limited, posing a future challenge.

**Table 4:** Current clinical trials for connective tissue disease (CTD) and interstitial lung disease (ILD) from the NIH National Library of Medicine database

Number of clinical trial	Study title	Status	Target disease	Intervention/treatment	Cell number	Number of doses	Dosage interval	Number of patients	Remarkable findings
NCT01873625	Transplantation of Bone Marrow Derived Mesenchymal Stem Cells in Affected Knee Osteoarthritis by Rheumatoid Arthritis	Completed	Rheumatoid arthritis	BM-MSCs transplantation	4.0×10^7^ cells/body	Single	-	60	Intra-articular knee implantation of MSCs appeared safe and well tolerated, with a trend toward clinical efficacy.
NCT03691909	Phase 1/2a Clinical Trial to Assess the Safety of HB-adMSCs for the Treatment of Rheumatoid Arthritis	Completed	Rheumatoid arthritis	Autologous ASCs	NA	Single	-	15	No serious adverse events; improved joint function in RA.
NCT03618784	Safety and Efficacy of FURESTEM-RA Inj. in Patients With Moderate to Severe Rheumatoid Arthritis	Completed	Rheumatoid arthritis	FURESTEM-RA injection	5.0×10^7^, 1.0×10^8^ cells/body	Three	4 weeks	33	ND
NCT03798028	The Safety and Effects of Mesenchymal Stem Cell (MSCs) in the Treatment of Rheumatoid Arthritis	Unknown	Rheumatoid arthritis	UC-MSCs	1.0×10^6^ cells/kg	Single	-	250	ND
NCT04971980	Safety and Efficacy Study of Human Umbilical Cord-Derived Mesenchymal Stem Cells(BC-U001) for Rheumatoid Arthritis	Recruiting	Rheumatoid arthritis	Human UC-MSCs infusion (BC-U001)	0.5, 1.0, 1.5 ×10^6^ cells/kg	Single	-	9	ND
NCT03333681	Evaluation of Stem Cell Therapy Effects on the Immune Response in Rheumatoid Arthritis Patients	Completed	Rheumatoid arthritis	Autologous MSCs	1.0 to 2.0 ×10^6^ cells/kg	Single	-	15	ND
NCT02633163	Phase 2 Trial of Mesenchymal Stem Cells in Systemic Lupus Erythematosus (MiSLE)	Recruiting	Systemic lupus erythematosus	Low- or high-dose MSCs	1.0, 5.0 ×10^6^ cells/kg	Single	-	81	ND
NCT03171194	Pilot Trial of Mesenchymal Stem Cells for Systemic Lupus Erythematosus	Completed	Systemic lupus erythematosus	Low-dose UC-MSCs	1.0×10^6^ cells/kg	Single	-	6	No serious adverse events; potential efficacy in lupus
NCT03562065	Treatment of Refractory Systemic Lupus Erythematosus by Allogeneic Mesenchymal Stem Cells Derived From the Umbilical Cord (MSC-SLE)	Recruiting	Systemic lupus erythematosus	Biological: MSCs	1.0, 2.0, 4.0×10^6^ cells/kg	Single	-	10	ND
NCT01539902	Phase 2 Study of Human Umbilical Cord Derived Mesenchymal Stem Cell for the Treatment of Lupus Nephritis (hUC-MSC-SLE)	Unknown	Lupus nephritis	UC-MSCs	NA	NA	NA	25	UC-MSC shows no additional effect beyond standard immunosuppression.
NCT05631717	The Study of Comparing the Efficacy and Safety of Human Umbilical Cord MSCs and Low-dose IL-2 in the Treatment of LN	Recruiting	Lupus nephritis/Systemic lupus erythematosus	Human UC-MSCs, drug: interleukin-2	1.0×10^6^ cells/kg	Single	-	40	ND
NCT03580291	Human Umbilical Cord Mesenchymal Stem Cells Treatment for Lupus Nephritis (LN)	Unknown	Lupus nephritis	MSCs, drug: mycophenolate Mofetil	2.0×10^6^ cells/kg	Single	-	230	ND
NCT04432545	Infusion of Allogeneic Mesenchymal Stem Cells in Patients With Diffuse Cutaneous Systemic Sclerosis With Refractory Pulmonary Involvement	Available	Systemic sclerosis / Pulmonary hypertension / Pulmonary fibrosis	MSCs from Wharton jelly, intravenous infusion	NA	NA	-	NA	ND
NCT03060551	Injection of Autologous Adipose-derived Stromal Vascular Fraction in the Finger of Systemic Sclerosis Patients	Completed	Systemic sclerosis	SVF injection	-	NA	-	18	Significant improvement in skin fibrosis, hand edema, and quality of life; 31.6% of ulcers healed at 24 weeks.
NCT03211793	Mesenchymal Stromal Cells as Treatment for Digital Ulcers in Systemic Sclerosis	Recruiting	Systemic sclerosis/digital ulcer	MSCs	5×10^7^ cells/body	Single	-	20	ND
NCT02213705	Treatment of Refractory Sever Systemic Scleroderma by Injection of Allogeneic Mesenchymal Stem Cells	Completed	Systemic scleroderma	Injection of allogeneic MSCs	NA	NA	-	20	ND
NCT00962923	Allogeneic Mesenchymal Stem Cells Transplantation for Systemic Sclerosis (SSc)	Unknown	Systemic sclerosis	Allogeneic MSCs	1.0×10^6^ cells/kg	Single	-	20	Feasible treatment; potential benefit in SSc.
NCT02975960	ADMSCs for the Treatment of Systemic Sclerosis	Completed	Systemic sclerosis	Injection of autologous SVF	-	-	-	7	ND
NCT01919827	Study of Autologous Mesenchymal Stem Cells to Treat Idiopathic Pulmonary Fibrosis	Completed	Idiopathic pulmonary fibrosis	Endobronchial infusion, autologous BM-MSCs	NA	NA	-	17	No treatment-related severe adverse events observed.
NCT02277145	A Study on Radiation-induced Pulmonary Fibrosis Treated With Clinical Grade Umbilical Cord Mesenchymal Stem Cells	Completed	Post-radiotherapy pulmonary fibrosis	UC-MSCs	1.0×10^6^ cells/kg	Single	-	10	ND
NCT03929120	Allogeneic Bone Marrow Mesenchymal Stem Cells for Patients With Interstitial Lung Disease (ILD) & Connective Tissue Disorders (CTD)	Completed	Interstitial lung disease/connective tissue diseases	Allogeneic BM-MSCs	0.5 to 1.0×10^6^ cells/kg	Single	-	10	ND
NCT02594839	Safety and Efficacy of Allogeneic Mesenchymal Stem Cells in Patients With Rapidly Progressive Interstitial Lung Disease	Completed	Idiopathic interstitial pneumonia/interstitial lung disease/idiopathic pulmonary fibrosis	BM-MSCs	NA	Two	Day 0,7	20	ND
NCT05468502	Phase I/IIa Clinical Trial of Human Umbilical Cord Mesenchymal Stem Cell Injection in the Treatment of Idiopathic Pulmonary Fibrosis (IPF)	Recruiting	Idiopathic pulmonary fibrosis	UC-MSCs injection	6.0×10^6^, 3.0×10^7^, 6.0×10^7^, and 9.0×10^7^ cells/person	Single	-	18	ND
NCT01385644	A Study to Evaluate the Potential Role of Mesenchymal Stem Cells in the Treatment of Idiopathic Pulmonary Fibrosis	Completed	Idiopathic pulmonary fibrosis	Placental MSCs	1.0, 2.0 ×10^6^ cells/kg	Two	3 months	8	Possibly favorable; one case of small bowel obstruction and all cases of halitosis.

*NA* not available, *ND* not described, *MSCs* mesenchymal stem cells, *BM-MSCs* bone marrow-derived MSCs, *UC-MSCs* Umbilical cord blood, *ASCs* Adipose-derived MSCs, *SVF* stromal vascular fraction, *RA* rheumatoid arthritis, *SSc* systemic sclerosis

## Conclusion

In conclusion, recent advances in the treatment of CTD have yielded a myriad of therapeutic options, particularly in the realm of RA and SLE. Although in this regard, conventional immunosuppressants and biological or targeted synthetic DMARDs have shown certain efficacy, challenges persist with respect to issues such as side effects and the lack of effective treatments for various CTDs, including SSc. CTD often manifests with organ damage, and ILD has emerged as a particular concern, given its significant impact on patient prognosis. Despite the current application of treatment modalities involving corticosteroids, immunosuppressive agents, and anti-fibrotic agents, CTD is still associated with a high incidence of respiratory failure and mortality.

In this context, MSCs have gain considerable attention with respect to their regenerative potential. Specifically, ASCs are gaining prominence on account of their accessibility and immunomodulatory effects. In this review, we have provided an overview of evidence obtained from preclinical studies that have evaluated the efficacy of MSC therapy in managing CTD and ILD, with a particular emphasis on AdSC therapy. The immunomodulatory effects of MSCs are highlighted, emphasizing their capacity to modulate a range of different immune cell types and cytokines, thereby identifying these stem cells as a promising avenue for CTD treatment. Notably, compared with other MSC types, such as BM-MSCs, ASCs have been established to have a number advantages in terms of accessibility, proliferation rates, and immunomodulatory potency.

Furthermore, our review of preclinical models, including those for RA, SLE, SSc, and ILD, highlight the potential of MSC/ASC therapy in ameliorating disease severity and improving patient outcomes. In addition, we also assess the challenges associated with the application ASC therapy, including intravenous administration-related complications. In response to these challenges, recent studies have begun the evaluate the efficacy of pharmacologically primed ASC therapy, introducing innovative approaches designed to enhance therapeutic effects. Among these, investigations into heparin-primed ASCs have yielded promising results in mitigating CTD-related symptoms.

We conclude the review by providing insights into the clinical applications of MSCs/ASCs for CTDs, including rheumatoid arthritis, systemic lupus erythematosus, systemic sclerosis, and interstitial lung disease. Clinical trials conducted to date have verified the safety and potential efficacy of MSCs, particularly ASCs, in treating refractory cases, paving the way for further research and development. In summary, the multifaceted potential of MSCs, particularly ASCs, in managing CTD and ILD represents a promising avenue for future therapeutic interventions. Moreover, the development of pharmacologically enhanced ASCs will provide innovative strategies for overcoming current challenges, thereby broadening the scope for advancing the field and improving patient outcomes.

## Data Availability

Not applicable.

## Abbreviations

MSCs: Mesenchymal stem cells
ASCs: Adipose-derived MSCs
CTD: Connective tissue disease
ILD: Interstitial lung disease
RA: Rheumatoid arthritis
DMARDs: Disease-modifying anti-rheumatic drugs
SLE: Systemic lupus erythematosus
CYC: Cyclophosphamide
MMF: Mycophenolate mofetil
SSc: Systemic sclerosis
HLA: Human leukocyte antigen
TGF-β1: Transforming growth factor-β1
PGE2: Prostaglandin E2
HGF: Hepatocyte growth factor
NK: Natural killer
TSG-6: TNF-α-stimulated gene 6
MMPs: Matrix metalloproteinases
mRNA: Messenger RNA
BM-MSCs: Bone marrow-derived MSCs
UC-MSCs: Umbilical cord blood-derived MSCs
HOCl: Hypochlorous acid
hepASCs: Heparin-enhanced ASCs
ACR: American College of Rheumatology
IPF: Idiopathic pulmonary fibrosis
